# Complete Genome Sequence of Aeromonas salmonicida subsp. *masoucida* Strain BR19001YR, Isolated from Diseased Korean Rockfish (Sebastes schlegelii)

**DOI:** 10.1128/MRA.01281-20

**Published:** 2021-01-21

**Authors:** Yu-Ra Kang, Ahran Kim, Yoonhang Lee, Nameun Kim, HeyongJin Roh, Do-Hyung Kim

**Affiliations:** a Department of Aquatic Life Medicine, Pukyong National University, Busan, Republic of Korea; b Pathology Division, National Fisheries Research and Development Institute, Busan, Republic of Korea; University of Maryland School of Medicine

## Abstract

We report the complete genome sequence of the virulent Aeromonas salmonicida subsp. *masoucida* strain BR19001YR, isolated from diseased black rockfish (Sebastes schlegelii). Sequencing of the circular chromosome and three plasmids using the PacBio and Illumina platforms yielded 4,982,192 bp with a 58.24% G+C content.

## ANNOUNCEMENT

Aeromonas salmonicida is an important bacterial fish pathogen causing furunculosis in many different freshwater and marine fish species worldwide (1). *A. salmonicida* subsp. *masoucida* BR19001YR was isolated from the kidneys, spleens, and ulcerated skin of diseased black rockfish showing skin ulcers and hemorrhages and/or exophthalmos. The isolate was inoculated onto tryptic soy agar (TSA) and incubated at 28°C for 48 h. Colonies identified as *A. salmonicida* subsp. *masoucida* by PCR (2) were stored in brain heart infusion broth supplemented with 15% glycerol at −80°C.

A single colony of *A. salmonicida* subsp. *masoucida* BR19001YR was inoculated into tryptic soy broth and incubated at 28°C for 48 h for DNA extraction. The bacterial culture was then resuspended in sterile saline (0.85% [wt/vol] NaCl), and the genomic DNA was extracted using the DNeasy blood and tissue kit (Qiagen, Germany) according to the manufacturer’s protocol. Whole-genome sequencing was performed with PacBio RS II (Pacific Biosciences, USA) single-molecule real-time (SMRT) technology. Genomic DNA was sheared with g-TUBE (Covaris, USA) into a 20-kb size and purified using AMPure PB magnetic beads (Beckman Coulter, USA). A Bioanalyzer 2100 instrument (Agilent, USA) was used to determine the actual size distribution. The library was prepared using a PacBio DNA template prep kit 1.0 and DNA/polymerase binding kit P6. The reads generated by the PacBio RS II were filtered and analyzed with PacBio SMRT Analysis v2.3.0, resulting in 127,719 reads (*N*_50_, 15,478 bp; mean subread length, 10,796 bp) with a G+C content of 56.12%. Illumina short-read sequencing was accomplished by randomly shearing the extracted genomic DNA to yield DNA fragments measuring an average of 350 bp using a Covaris S2 ultrasonicator. Library preparation was performed following the Illumina TruSeq DNA PCR-free preparation kit according to the manufacturer’s instructions. The final library size and quality were evaluated electrophoretically with an Agilent high-sensitivity DNA kit. The 150-bp paired-end reads were sequenced on the Illumina NovaSeq 6000 platform, resulting in a total of 35,962,872 reads with a Q30 of 92.00% and G+C content of 58.57%. CANU v1.7 (3) and Pilon v1.22 (4) were used for *de novo* assembly. When both ends of the contig overlapped, the contig was regarded as a circular form, and overlapped regions were manually trimmed. The BR19001YR genome yielded a total size of 4,982,192 bp encompassing four contigs with a G+C content of 58.24% and a total of 4,841 genes. The total number of genes in the BR19001YR genome was annotated by the NCBI Prokaryotic Genome Annotation Pipeline (PGAP) v4.13 (5). Default parameters were used for all the software used in this study.

The results show that this genome harbored a circular chromosome measuring 4,780,847 bp with 4,627 protein-coding sequences (CDSs). The genome of BR19001YR contains 124 tRNA and 31 rRNA genes. It also contained three plasmids, pBY1 (66,892 bp), pBY2 (70,953 bp), and pBY3 (63,500 bp), containing 79, 70, and 65 CDSs, respectively. The average nucleotide identity (ANI) (6) analysis using publicly available *A. salmonicida* strains showed that the strain BR19001YR shared 99.83% to 99.9% identity with strains of *A. salmonicida* subsp. *masoucida* (*n* = 5). Interestingly, only strain BR19001YR harbored pBY3, containing the P-type type IV secretion system, and *catB7*, *sul2,* and *tet*(A) and *tet*(Y), which are related to resistance against chloramphenicol, sulfa, and tetracycline drugs, respectively. This plasmid showed 98.79% to 99.93% nucleotide identity with a plasmid of some bacterial fish pathogens, such as p3PS10 (GenBank accession number CP013819.1) of Piscirickettsia salmonis, pVb1978 (accession number MG456577.1) of Vibrio alginolyticus, and pEIB202 (accession number CP001136.1) of Edwardsiella tarda EIB202. However, the sequence coverages between pBY3 and each above-mentioned plasmid were only 55%, 54%, and 57%, respectively, indicating that pBY3 is a very distinct plasmid. This new genome sequence can be used for further studies in the future to elucidate the pathogenicity and host specificity of highly virulent *A. salmonicida* subsp. *masoucida*.

### Data availability.

The genome sequence of *A. salmonicida* subsp. *masoucida* strain BR19001YR has been deposited in GenBank under the accession numbers CP060030.1, CP060031.1, CP060032.1, and CP060033.1. The Illumina raw reads (SRX8980909) and PacBio raw reads (SRX8380278) have been submitted to the NCBI Sequence Read Archive (SRA) database under BioProject number PRJNA609824.
